# Comparative evaluation of echocardiography indices during the transition to extrauterine life between small and appropriate for gestational age infants

**DOI:** 10.3389/fped.2022.1045242

**Published:** 2023-01-16

**Authors:** Laura Mihaela Suciu, Regan E. Giesinger, Claudiu Mărginean, Mihai Muntean, Manuela Cucerea, Amalia Făgărășan, Patrick McNamara

**Affiliations:** ^1^ Department of Pediatrics, University of Medicine Pharmacy Science and Technology George Emil Palade of Târgu Mureș, Târgu Mureș, Romania; ^2^ Division of Neonatology, Department of Pediatrics, University of Iowa Stead Family Children's Hospital, Iowa, IA, United States; ^3^ Department of Obstetrics and Gynecology, University of Medicine Pharmacy Science and Technology George Emil Palade of Târgu Mureș, Târgu Mureș, Romania

**Keywords:** small for gestational age, postnatal transition, targeted neonatal echocardiography, pulmonary vascular resistance, heart function

## Abstract

**Objectives:**

To study changes in heart function and hemodynamics during the transitional period in small for gestational (SGA) infants and appropriate (AGA) healthier counterparts.

**Design:**

A hospital based prospective observational study was performed at a perinatal center. Echocardiograms were performed on the first postnatal day and again at 48 h age. Term SGA infants were compared with those AGA newborns matched for the GA and mode of delivery.

**Results:**

Eighteen SGA infants were compared with 18 AGA infants [gestation 38 ± 1.5 vs. 38 ± 1.2 weeks, *p* > 0.05 and birthweight 2331 ± 345 vs. 3332 ± 405 grams, *p* < 0.05, respectively]. Maternal weight and body mass index was higher among non-affected pregnancies, 61% infants were born vaginally, and no differences in cord blood pH at birth were noted. SGA infants had higher systolic and mean blood pressure at both time points, lower indices of right ventricular (RV) performance [TAPSE (tricuspid annular peak systolic excursion) 7.4 ± 2.8 vs. 9.3 ± 0.7 on day 1, 7.2 ± 2.8 vs. 9.2 ± 0.5 on day 2, *p* = 0.001], lower pulmonary acceleration time (PAAT) suggestive of elevated pulmonary vascular resistance [56.4 ± 10.5 vs. 65.7 ± 13.2 on day 1, 61.4 ± 12.5 vs. 71.5 ± 15.7 on day 2, *p* = 0.01] and higher left ventricular (LV) ejection fraction [62.1 ± 7.8 vs. 54.9 ± 5.5 on day 1, 61.9 ± 7.6 vs. 55.8 ± 4.9 on day 2, *p* = 0.003].

**Conclusions:**

SGA infants had evidence of higher pulmonary vascular resistance, and lower RV performance during the postnatal transition. The relevance and impact of these changes to hemodynamic disease states during the postnatal transition requires prospective investigation.

## Introduction

Fetuses with an estimated fetal weight (EFW) less than 10th percentile are at increased risk of adverse outcome such intrauterine death (1), preterm birth (2), and meconium-stained amniotic liquid (3). After birth, there is a greater risk of neurological and neurodevelopmental morbidity (4), and a 10-to-30-fold increase in the risk of developing cerebral palsy (5). In a multivariable analysis of the temporal trends (2010–2019) in hypoxic ischemic encephalopathy (HIE) and peripartum risk factors, small for gestational age neonates were found to be associated with increased odds of HIE (6). Recent studies conclude that the *fetus* with intrauterine growth retardation (IUGR) is at greater risk of abnormalities of cardiac size and shape (7), arterial and ventricular wall thickness and cardiac dilatation beyond neonatal period (8), metabolic abnormalities (9), and permanently altered autonomic cardiovascular control (10). In chronic hypoxemia, IUGR *fetuses* redistribute cardiac output to maximize the oxygen and nutrient supply to the brain. The fetal circulation is a parallel circuit where most of the right ventricular (RV) output is shunted through the ductus arteriosus (DA) to the descending aorta, and the left ventricular (LV) output mainly supplies the upper body and brain. Vasoconstriction of the peripheral vascular bed (11) and vasodilation of the cerebral arteries result in a preferential shift of cardiac output towards the brain (12, 13).

After birth, experimental studies on animals demonstrated a progressive decrease in pulmonary vascular resistance (PVR) (14), concomitant with the switch in the direction of flows at the level of DA and patent foramen ovale (PFO) and, shortly after that, the closure of transitional shunts without any discernible changes in heart function during the first postnatal day in healthy term neonates (15, 16). Failure of the normal postnatal decrease in PVR immediately after birth results in continued right-to-left shunting across the fetal shunts, and persistence of elevated pressure in the pulmonary circulation (17). In a recent study, Young et al. (18) demonstrated that IUGR is a major risk factor for chronic lung disease and pulmonary hypertension (PH). In addition, Khemani et al. demonstrated that PH in the setting small for gestational age (SGA) was associated with worse survival rates amongst a preterm population (19). Previous studies also reported that IUGR was found in 40% of patients with BPD and PH and is therefore an important risk factor for screening (20). Characterization of the changes in heart function and hemodynamics during the transitional period in SGA infants is a knowledge gap and important for appraising disease susceptibility. In this study, we hypothesized that SGA newborns will display modified indices of pulmonary vascular and heart development during the transitional period.

## Material and methods

We conducted a prospective cohort study of pregnant women detected during the first to second trimester of pregnancy with EFW < 10th centile and group of matched for gestational age (GA) healthy pregnant (control group). This study was performed at a Level II neonatal intensive care unit (NICU) at Maternity Department of County Hospital, Târgu Mures Romania between June 2021, and January 2022. The total annual birth rate in this center is approximately 1,500 cases; of these, 5% are SGA newborns leading to 75 potential eligible infants/year. Pregnant women during the first to second trimester of pregnancy, in whom EFW < 10th centile, were examined closely during the third trimester of pregnancy as part of routine care until spontaneous labor or delivery. A control group, consisting of healthy pregnant women with EFW between 10 and 90th centile, were matched one-to-one according to GA. Mothers were approached again at term (37–41 weeks) and asked to participate in the study. The informed consent process took place before active labor, and only those who agree to participate and signed a consent form were included. The study was approved by the Ethics of Research of University of Medicine, Pharmacy Science, and Technology George Emil Palade of Targu Mures, Târgu Mureș, Romania (Institutional Review Board Number 1241/14.01.2021). Pregnancies where absent or reversed diastolic flow on UA and MCA Doppler was detected were excluded, as these babies usually are delivered prematurely.

### Concealment and blinding

The health care providers who attend the pregnant women at birth were blinded to the subject's participation into the study. In addition, data abstractors were blinded to group allocation. To minimize operator dependency error, the maternal scans were performed by a single experienced obstetrician (C.M) and the neonatal scans were performed by a single sonographer (L.M.S). The sonographers were not blinded to group allocations and echo order; however, all 72 neonatal scans used for this study were de- identified, digitally stored and all the measurements were performed off-line in a random order at the completion of recruitment. To ensure blinding, clinical data were collected separately and were merged with echocardiography data prior to statistical analysis.

Eligibility Criteria:

SGA cases were included if they satisfied the following eligibility criteria:

(i) Birth Weight (BW) ≤ 10th percentile on Fenton growth charts
(ii) Evidence of EFW ≤ 10th percentile in first to second trimester
(iii) Born after 37 completed weeks GA and clinically asymptomatic
(iv) Singleton pregnancy with documented GA based on the LMP date and first trimester ultrasound exam

Appropriate for Gestational Age (AGA) controls were included if they satisfied the following eligibility criteria:

(i) AGA infant with BW > 10–90th centile on Fenton growth charts at birth
(ii) Evidence of EFW > 10th centile in first to second trimester
(iii) Low-risk singleton pregnancy with documented GA based on the LMP date and first trimester ultrasound exam and were followed up until spontaneous or induced labor and delivery
(iv) Born at term gestation and clinically asymptomatic
(v) All eligible fetuses had documented umbilical artery (UA) and middle cerebral artery (MCA) Doppler tracings prior to birth

Exclusion criteria were as follows:

(i) Maternal age < 18 years
(ii) Fetuses with structural anomalies.
(iii) Infants detected with genetic or dysmorphic abnormalities
(iv) Infants born less than 37 weeks GA
(v) Evidence of perinatal hypoxia-ischemia (arterial cord blood pH < 7, 5-min Apgar score < 7, and/or need for bag and mask ventilation)
(vi) Evidence of significant congenital heart disease [except patent foramen ovale (PFO), patent ductus arteriosus (PDA)]

EFW was calculated from measurements of the biparietal diameter, head circumference, abdominal circumference and femur length using Hadlock formula (21, 22). Subsequently UA and MCA PI were measured. Doppler analyses were performed for all study participants using the same ultrasound machine (Voluson GE Model) and by the same physician. Pulsatility index (PI) = peak systolic velocity -end diastolic velocity/ time averaged maximum velocity. For this study, the last ultrasound examination prior to delivery was used for the analysis. Per convention, the UA Doppler waveform was recorded in free loop, while MCA Doppler waveform was recorded as close possible to the vessel originating from the circle of Willis.

Arterial cord blood pH measurement after birth was standard of care for all newborns in our institution; specifically, blood analysis was performed with I-STAT Analyzer (MN:300-G, Abbot Point of Care Inc). Immediately after birth, the clinical status of newborn infants was evaluated by the attending neonatologist according to the Apgar score at 1 and 5 min. Other data including birthweight, cranial perimeter and length were assessed.

Arterial blood pressure was measured at two different time points (24- and 48-h postnatal age), prior to each echocardiographic evaluation exam was performed, using a noninvasively oscillometric method (B40 Monitor, GE Medical System Information Technologies, Inc Milwaukee, WI, USA) with the infant in a calm state. An appropriate size cuff for the arm was used. An average of two consecutive measurements were included in analysis. We choose to report this parameter because it is widely used to define hypotension; however, there are a number of factors that can affect interpretation (23, 24).

Peripheral Oxygen saturation (SpO_2_) was measured because several studies reported SpO_2_ screening of critical congenital heart disease (CCHD) to be feasible and cost effective (25), and also has been found to reduce missed diagnosis of CCHD (26, 27).

### Echocardiography Assessment

Paired echocardiography evaluations were performed at 24-h (Echo 1) and 48-h (Echo 2) postnatal age. These two time points were specifically chosen because healthy term newborns are usually discharged after 2 complete days of hospitalization. Exams were performed with the Versana active ultrasound system using a 12-MHz neonatal transducer (GE Medical System, Milwaukee, Wisconsin). Each echocardiogram was performed with the infants in the supine position, either asleep or in a resting state, without prior sedation, and lasted less than 40 min. Small volumes (2–5 ml) of Glucose 10% were administered by mouth and/or swaddling techniques to help the infant reach a quiet state. Image acquisition was conducted in accordance with the guidelines for targeted neonatal echocardiography (28). Comprehensive evaluation of heart function, pulmonary and systemic hemodynamics, shunt hemodynamics were performed according to a standardized protocol; specific details of echocardiography indices are described in Supplementary Appendix Table S1. Five consecutive cardiac cycles were recorded and for pulse-wave Doppler (PWD) measurements three representative waveforms were measured, averaged, and included in the analysis. Data on flow velocities, velocity time integral (VTI) and heart rate were analyzed. The values of peak systolic velocity (PS), end-diastolic velocity (ED) and V mean (traced), were recorded, and included in the analyses. Resistive index (RI) was calculated according to the formula RI = (Vs-Vd)/Vs. Pulsatility index was calculated according to formula PI = (Vs-Vd)/V mean.

### Specific echocardiography measurements

Pulmonary Acceleration Time (PAAT) and Right Ventricular Ejection Time (RVET) were measured from Doppler spectral flow velocity envelope obtained by placing a pulsed Doppler sample volume at the pulmonary valve annulus. PAAT is defined as the interval between the onset of systolic pulmonary arterial flow and peak flow velocity. RVET is measured from the interval between the onset of RV ejection to the point of systolic pulmonary arterial flow cessation. Pulmonary Vascular Resistance index (PVRi) was calculated by dividing RVET to PAAT to account for the impact of heart rate of time-dependent indices. Mean PAAT and RVET, from three well-defined waveforms, were used for data analysis. A RVET: PAAT ratio > 4 was considered abnormal (29). *Right ventricular systolic function* was assessed using both tricuspid annular peak systolic excursion (TAPSE) and fractional area change (FAC) measured from the RV 3-chamber view. Abnormal RV systolic function was defined be TAPSE <8 mm (30) and FAC < 35% (23) based on normative data in the transitional period. *Left ventricular systolic function* was assessed by calculating ejection fraction using the Simpson biplane method. A threshold LV-EF (%) value <55% was used as a threshold abnormal LV systolic function (29) Diastolic RV and LV function was assessed by measuring the ratio of the peak velocities of early (E) and late (A) diastolic inflow across the respective atrioventricular valve. A ratio of E:A < 1.0 has been associated with poor compliance of the corresponding ventricle, denoting altered diastolic function (31). In addition, LV isovolumic relaxation time (IVRT) was obtained from the apical 4-chamber view using pulse wave Doppler with the sample gate placed at the level of the tips of mitral valve leaflets. A IVRT value higher than 50 msec was considered representative of abnormal LV diastolic function (32).

### Outcomes

The *primary outcome* was pulmonary vascular resistance index (PVRi = RVET: PAAT), an echocardiography parameter of the pulmonary vascular disease. *Secondary outcomes* included echocardiography indices of pulmonary hemodynamics (PAAT, RVET, end-systolic eccentricity index, right ventricular systolic pressure), LV systolic performance [shortening fraction (SF), ejection fraction (EF), LV output (LVO)], RV systolic performance [RV output (RVO), RV fractional area change (FAC), and tricuspid annular peak systolic velocity (TAPSE)], left heart diastolic function [left atrial to aortic root ratio (LA: Ao); pulmonary vein peak systolic and diastolic velocities; mitral E and A velocities, E:A ratio, isovolumic relaxation time (IVRT)] and systemic hypoperfusion [celiac, superior mesenteric and middle cerebral artery peak systolic and diastolic velocity].

### Statistics

Values were presented as mean (standard deviations) unless stated otherwise. Independent sample T test was used to assess the differences between SGA and AGA groups. Continuous data were reported as mean (SD) and categorical data are presented as n (%). Changes in variables with repeated measurements were assessed using two-way repeated measures analysis of variance. A *p* value less than 0.05 was considered statistically significant. We used SPSS 21(Chicago, IL) to conduct the analysis. A predefined sample size of 25 pregnant women was selected for convenience purposes.

## Results

**Figure 1:**
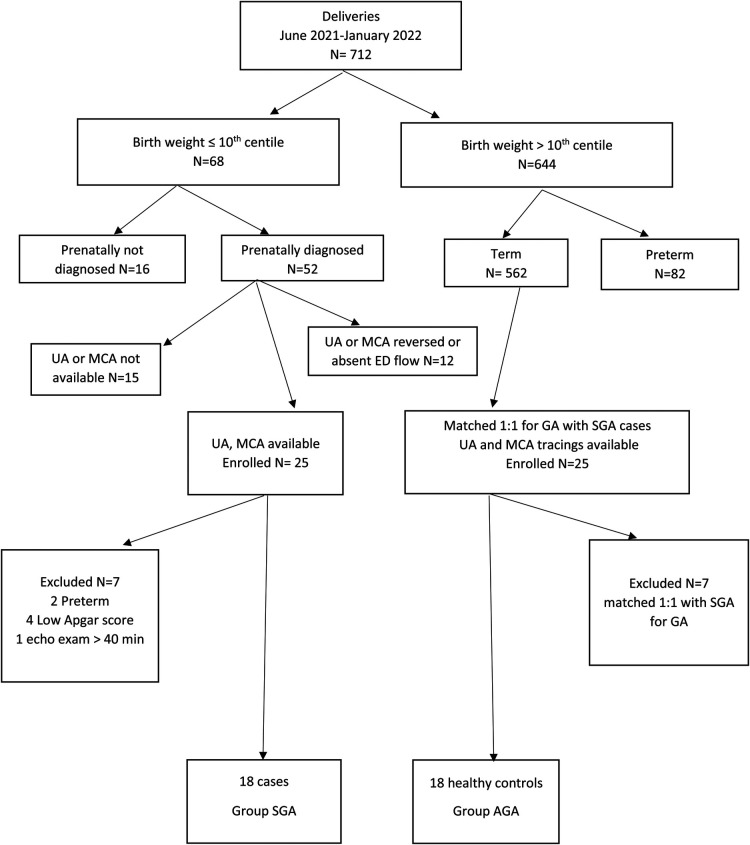
Recruitment flowchart for study participants.

In total, 25 pregnant women were serially assessed for eligibility during the study period and closely examined during the third trimester of pregnancy. Subsequently, 7 newborns with low Apgar score, preterm birth, and prolonged echocardiography examination were excluded. Ultimately, 36 patients were allocated into two groups of 18 cases (SGA Group) and controls (AGA Group). (Figure 1) The final postnatal cohort for echocardiography evaluation consisted of 36 infants (16 males), whose mean (SD) GA and weight at birth were 38 ± 1.3 weeks and 2.8 ± 0.6 kg respectively. Twenty-two (62%) infants were born vaginally. None of the studied patients required admission to the NICU.

***Maternal baseline characteristics*** (Table 1): Maternal weight and body mass index were higher among non- affected pregnancies. Umbilical artery Doppler pulsatility index was lower and middle cerebral artery pulsatility index higher among affected pregnancies (*p* < .001).

**Table 1:** Demographic indices of the SGA infants and AGA controls.

Variable	SGA group *n* = 18	AGA group *n* = 18	*p* value (Group)
***Maternal characteristics***			
Maternal age	25.2 (7.2)	28.2 (6.1)	0.85
Maternal height, cm	160.6 (5.7)	161.1 (4.8)	0.75
Maternal weight, kg	63.8 (11.3)	74.6 (12.5)	0.01
Maternal BMI, Kg/m^2^	24.7 (4.3)	28.7 (4.4)	0.01
Maternal smoking	5 (27.8)	7 (38.8)	0.49
Maternal nulliparity, %	10 (55.6)	5 (27.7)	0.09
Maternal diabetes	0	1 (5.5)	0.32
Maternal hypertensive	0	2 (11.1)	0.15
**Mode of delivery, %**			
Vaginal	11 (61)	11 (61)	0.9
Cesarean	7 (39)	7 (39)	
***Fetal Characteristics***			
Gestational age at enrollment, weeks	32 (1)	31(2)	0.4
Fetal MCA PI	1.5 (0.2)	1.1 (0.2)	<.001
Fetal UA PI	1.1 (0.2)	2.1 (0.3)	<.001
***Neonatal Characteristics***			
Gestational age at delivery, weeks	38 ± 1.5	38 ± 1.2	0.41
Birth weight, grams	2331 ± 345	3332 ± 405	<.001
Birth weight percentile	2.1 (2.6)	46.6 (26.1)	<.01
Birth weight < 3rd centile	14 (77.8)	–	<.001
Head circumference, cm	31 ± 1.8	33 ± 1.4	<.001
BSA	0.17 ± 0.01	0.22 ± 0.01	<.001
Male sex, *n*	8 (44.5)	8 (44.5)	0.9
Apgar score at 5 min	8 (8–9)	9 (8–10)	.09
Apgar score at 10 min	9 (9–10)	9 (9–10)	0.27
Cord blood pH	7.2 (0.1)	7.2 (0.1)	0.15

BMI, body mass index; MCA, middle cerebral artery; UA, umbilical artery; PI, pulsatility index. Data are presented as mean ± SD, median (range) or frequency (percentage). *P* values are the result of independent-samples *T* test with significance set at *p* < 0.05.

***Neonatal cardiorespiratory variables*** (Table 1): Neonatal birthweight, head circumference and body surface area were lower among SGA group (*p* < .05) but no differences in APGAR score at 1- and 5-min and cord pH at birth were noted. Systolic and mean blood pressure were higher in SGA infants at both time points (*p* < 0.05), but no intergroup differences in either pre- or post -ductal oxygenation were noted (Table 2).

**Table 2:** Effects of group and time on preductal blood pressure and pre- and postductal oxygen saturation.

	SGA Group *n* = 18		AGA Group *n* = 18		*p* value (Time)*	*p* value (Group)^#^
	*Day 1*	*Day 2*	*Day 1*	*Day 2*		
Systolic BP (right arm), mmHg	69.8 (5.1)	73.1 (4.9)	55.2 (4.8)	58.5 (3.7)	0.001	0.001
Diastolic BP (right arm), mmHg	38.1 (3.1)	40.8 (3.7)	36.9 (3.1)	38.2 (3.7)	0.001	0.07
Mean BP (right arm), mmHg	48.3 (3.6)	51.2 (3.1)	43.5 (4.61)	44.8 (2.9)	0.003	0.001
Oxygen Saturation (right arm), %	98.3 (0.7)	98.7 (0.8)	98.8 (0.5)	98.8 (0.5)	0.05	0.09
Oxygen Saturation (foot), %	98.3 (1.1)	98.8 (0.9)	98.9 (0.5)	99.6 (0.5)	0.02	0.18

BP, blood pressure. Values are presented as mean (SD). *P* values are the results of general linear model repeated measures.

*P value (time) represents the significance of changing values over the two different time points.

^#^ *P* value (group) represents the significance of the difference between the two groups.

***Right ventricular function*** (Tables 3, 4): Indices of RV systolic function were lower in SGA infants (*p* < 0.05); specifically, RV-FAC and TAPSE were lower at both time-points. The rate of abnormal TAPSE or RV-FAC, suggestive of RV systolic dysfunction were 44% and 27.8% respectively in SGA infants. None of the AGA group reached abnormal thresholds of RV systolic function. In addition, tricuspid E/A ratio was also lower in the SGA group compared to AGA infants (*p* < 0.05). Although the incidence of abnormal tricuspid E/A ratio <1 was higher, this did not reach the statistical significance (*p* > 0.05).

**Table 3:** Effect of group and time on ventricular function.

	SGA Group *n* = 18		AGA Group *n* = 18		*p* value (Time)*	*p* value (Group)^#^
	*Echo 1*	*Echo 2*	*Echo 1*	*Echo 2*		
**Right ventricle function**						
Tricuspid E (cm/s)	43 (9)	41 (14)	49 (11)	46 (9)	0.22	0.07
Tricuspid A (cm/s)	57 (9)	55 (17)	62 (13)	59 (9)	0.36	0.25
Tricuspid E/A	0.7 (0.1)	0.6 (0.2)	0.8 (0.1)	0.8 (0.1)	0.04	0.03
RV ESA-3C (cm^2^)	3.4 (0.4)	3.5 (0.6)	3.7 (0.5)	4.1 (0.5)	0.004	0.011
RV EDA-3C (cm^2^)	5.4 (0.5)	5.6 (0.8)	6.3 (0.7)	6.6 (0.7)	0.02	0.001
FAC-3C, %	36.9 (2.2)	37.3 (2.1)	40.5 (0.8)	38.5 (0.9)	0.03	0.001
TAPSE	7.4 (2.8)	7.2 (2.8)	9.3 (0.7)	9.2 (0.5)	0.71	0.001
**Left ventricle function**						
PV D wave (cm/s)	45 (27)	42 (21)	53 (18)	42 (9)	0.01	0.08
PV S wave (cm/s)	51 (27)	43 (21)	63 (16)	52 (9)	0.07	0.47
Mitral E (cm/s)	47 (14)	45 (18)	59 (10)	54 (10)	0.16	0.01
Mitral A (cm/s)	48 (14)	44 (16)	59 (7)	53 (6)	0.06	0.001
Mitral E/A	0.9 (0.2)	0.8 (0.3)	1.1 (0.1)	1.1 (0.1)	0.74	0.09
IVRT (ms)	47.5 (9.3)	48.1 (15.4)	38.6 (4.4)	42.4 (3.3)	0.34	0.002
SF (%)	39.3 (11.6)	34.8 (10.7)	37.8 (4.1)	34.4 (13.5)	0.13	0.68
EF (%)-Simpson's biplane	62.1 (7.8)	61.9 (7.6)	54.9 (5.5)	55.8 (4.9)	0.9	0.003

E, early; A, atrial; 3 C, three chamber view; RV ESA, right ventricle end systolic area; RV EDA, right ventricle end diastolic area; FAC, fractional area change; TAPSE, tricuspid annular systolic peak excursion; PV, pulmonary vein; EF, ejection fraction; SF, shortening fraction; IVRT, isovolumic relaxation time. Values are presented as mean (SD).

*P value (time) represents the significance of changing values over the two different time points.

^#^ *P* value (group) represents the significance of the difference between the two groups.

**Table 4:** Frequency of abnormal hemodynamic thresholds for SGA infants compared to term neonates during transitional period.

	SGA Group *n* = 18		AGA Group *n* = 18		*p* value (Time)*	*p* value (Group)^#^
	*Day 1*	*Day 2*	*Day 1*	*Day 2*		
**Abnormal Right Heart Thresholds**						
PVRi > 4	6 (33.3)	4 (22.3)	2 (11.2)	1 (5.5)	0.77	0.47
Tricuspid E: A < 1	17 (94)	16 (88)	16(88.9)	14(77.8)	0.57	0.56
RV FAC-3C < 35%	5(27.8)	4 (22.2)	0	0	0.71	0.002
TAPSE < 8 mm	8 (44.4)	8 (44.4)	0	0	0.9	0.001
**Abnormal Left Heart Thresholds**						
IVRT > 50 msec	1 (5)	5 (22)	0	0	0.07	0.04
Mitral E: A < 1	8 (44)	10 (56)	5 (28)	4 (22)	0.38	0.06
EF (%)-Simpson's biplane < 55	4 (22.2)	3 (16.6)	2 (11.1)	1 (5.5)	0.26	0.03

PVRi, pulmonary vascular resistance index; RV-FAC-3C, right ventricle fractional area changes in three chamber view; TAPSE, tricuspid annular peak excursion; LV-EF, left ventricle ejection fraction. Values are presented as numbers and percentages. *P* values are the results of general linear model repeated measures.

*P value (time) represents the significance of changing values over the two different time points.

^#^ *P* value (group) represents the significance of the difference between the two groups.

***Left ventricular function*** (Tables 3, 4): Although SGA infants had higher mean value of EF (*p* < 0.01), 22% satisfied the criteria for LV systolic disfunction (EF < 55%). Indices of LV diastolic function were also different between groups; specifically, IVRT was longer and mitral valve peak E and A wave velocity was lower in SGA infants. No difference in mitral E/A ratio, however, was noted (*p* > 0.05). A strong trend towards higher rate of abnormal mitral E/A peak velocity ratio <1.0 and IVRT >50 msec were noted in SGA group (*p* = 0.06).

***Pulmonary and systemic hemodynamics*** (Tables 4, 5): SGA infants had longer PVRi (*p* < 0.01), lower RVO (*p* < 0.05) and higher rate of abnormal PVRi (>4) threshold compared to AGA infants. Similarly, the rates of bidirectional atrial and PDA shunt were higher in SGA infants at both time-points (*p* < 0.01 vs. time and group) indicating different loading condition of the RV in the SGA group (Table 6). Although SVRi was lower (*p* < 0.01), no differences in LVO were noted. No differences in systemic blood flow velocities, resistance and pulsatility indices were noted between groups over time (Supplementary Appendix Table S2).

**Table 5:** Effect of group and time on pulmonary and systemic hemodynamics.

	SGA Group *n* = 18		AGA Group *n* = 18		*p* Value (Time)*	*p* Value (Group)^#^
	*Echo 1*	*Echo 2*	*Echo 1*	*Echo 2*		
**Right ventricle hemodynamics**						
PAAT, ms	56.4 (10.5)	61.4 (12.5)	65.7 (13.2)	71.5 (15.7)	0.02	0.01
RVET, ms	208.3 (13.3	211.1 (13.7)	210.5 (34.8)	211.8 (35.1)	0.64	0.84
PVRi = RVET/PAAT	3.8 (0.7)	3.5 (0.5)	3.2 (0.7)	3.1 (0.5)	0.03	0.008
PA VTI (cms)	11.2 (1.7)	12.1 (2.3)	12.1 (1.3)	12.4 (1.6)	0.05	0.24
Heart rate	114.1 (15.9)	118.7 (18.7)	120.9 (11.5)	118.1 (11.6)	0.72	0.48
RVO (ml/kg/min)	163.4 (66.9)	191.43(75.9)	211.1 (47.8)	224.5 (58.4)	0.01	0.04
EI end systole = D1/D2	0.9(0.1)	0.9 (0.1)	0.9(0.1)	1.1 (0.1)	0.04	0.16
**Left ventricle hemodynamics**						
AoAcT	51.1 (4.8)	52.1 (4.8)	44.6 (6.1)	47.1 (5.4)	0.01	0.002
LVET	214.4 (18.3)	208.4 (19.3)	204.5 (15.8)	197.8 (13.1)	0.01	0.06
SVRi = LVET/AoAcT	4.2 (0.4)	3.9 (0.3)	4.6 (0.6)	4.2 (0.5)	0.001	0.04
Aortic VTI (cms)	12.5 (1.7)	13.1 (1.7)	10.8 (0.7)	12.9 (1.4)	0.001	0.05
Heart rate	115.6 (13.1)	122.6 (15.6)	125.5 (11.5)	116.5 (9.1)	0.61	0.61
LVO (ml/kg/min)	190.7 (36.3)	211.1 (39.2)	193.5 (24.7)	180.3 (20.8)	0.35	0.15
LA: Ao	1.4 (0.7)	1.2 (0.5)	1.4 (0.2)	1.1 (0.5)	0.04	0.81

PAAT, pulmonary acceleration time; RVET, right ventricular ejection time; PVRi, pulmonary vascular resistance index; AoAcT, aortic acceleration time; LVET, left ventricle ejection time; SVRi, systemic vascular resistance index; VTI, velocity time integral; HR, heart rate; EI, eccentricity index; Values are presented as mean (SD).

*P value (time) represents the significance of changing values over the two different time points.

^#^ *P* value (group) represents the significance of the difference between the two groups.

**Table 6:** Effect of group and time on transitional shunts.

	SGA Group *n* = 18		AGA Group *n* = 18		*p* value (Time)	*p* value (Group)
	*Echo 1*	*Echo 2*	*Echo 1*	*Echo 2*		
**Foramen ovale**						
Open overall	18 (100)	17 (94.4)	17 (94.4)	16 (88.9)		
Small restrictive with L–R shunt	15 (83)	10 (61)	13 (72.2)	15 (83.3)	0.33	0.31
Bidirectional shunt	3 (17)	7 (39)	4 (22.2)	1 (5.6)	0.001	0.005
**Ductus arteriosus**						
Open overall	14 (78)	6 (33)	16 (88.9)	5 (27.8)		
Small restrictive with L–R shunt	9 (50)	6 (33)	14 (77.8)	5 (27.8)	0.006	0.79
Bidirectional shunt	5 (28)	0	2 (11.1)	0	0.001	0.007

PFO, patent foramen ovale; PDA, patent ductus arteriosus. No patient had unrestrictive left to right shunt and pure right to left shunt. NS = *p* > 0.05; *p* values are the results of general linear model repeated measures. Values are presented as numbers and percentages.

## Discussion

In a prospective cohort of 18 term infants with an antenatal diagnosis of SGA we demonstrated higher systolic and mean blood pressure in the first 48 postnatal hours. In addition, SGA infants had evidence of higher PVR, lower RV systolic performance and more abnormal LV diastolic function.

Characterizing the normal postnatal cardiovascular adaptative changes is important to understand mechanism of illness and their relationship to illness severity. To date, knowledge is limited to preclinical studies. In a model of FGR affected preterm lambs, reduced LV output, higher systemic vascular resistance (SVR), and a lesser drop in PVR after birth were noted compared to AGA counterparts (33–35). In the setting of human FGR, SGA preterm had higher LV dimensions and LV output immediately after birth and were less able to increase LVO in the 1st four days after birth (36). These findings are consistent with Fouzas (37) et al., who demonstrated higher LV stroke volume and signs of LV diastolic dysfunction but no differences of the LV myocardial performance among an FGR affected population. On the contrary, Sehgal et al. (38) demonstrated impaired LV myocardial performance and lower arterial compliance among term SGA affected newborns evaluated on days 2 and 5 compared to healthier AGA. Both animal experimental and human natural history transitional studies demonstrated a progressive fall of PVR over the first 48–72 h after birth. In our cohort we demonstrated higher pulmonary artery acceleration time and lower RV output among SGA infants. Pulmonary artery acceleration time is a reliable marker of PVR and was previous validated among older children (39) and adults (40). Given the major changes in heart rate in the transitional period, indexing PAAT is an important consideration. In addition, persistent bidirectional flow across the PDA and PFO is further evidence of the differential adaptive changes in PVR. Our results are consistent with the findings of Sehgal et al. (41), who demonstrated higher baseline PVR indices before surfactant replacement and a lesser drop of PVR after surfactant among SGA affected preterm newborns compared to AGA controls. The discordance in magnitude of decline in PVR after birth in SGA infants, in the absence of primary lung disease, is noteworthy. It is plausible that pulmonary vasculature is subjected to arterial wall remodeling as noted in systemic arteries (42–44) or altered production of endogenous pulmonary vasodilators (PGI2 and bradykinin) (45, 46). These changes place the IUGR infant at higher risk of pulmonary vascular disease, particularly during the transitional period.

In the present study the systolic but not diastolic BP was higher among SGA neonates. The study by Fouzas et al. (37) showed differences in BP between SGA and AGA infants on postnatal day 2, whereas Zanardo et al. (47), which shows higher systolic BP but not diastolic BP among a formerly SGA population evaluated at 28-month of age. Other authors have demonstrated higher systolic and diastolic BP in newborns (48, 38), infants (49), and adults (50) of formerly affected SGA fetuses. The relationship between SGA population and higher BP may related to the known association with early endothelial dysfunction, impaired arterial vasodilation, and aortic wall intimal media thickening (aIMT) occurring *in utero*. The exact mechanisms of these associations are yet unknown, but evidence from recent studies indicates that impaired growth *in utero* triggers an adaptative process of arterial wall remodeling caused by increased pressure on fetal circulation in context of placental insufficiency (42–44). *Second*, chronic fetal *tissue hypoxia* itself induces proliferation of vascular smooth muscle and the adventitial fibroblast of precapillary vessels (51). After birth, the thickened arteries will be responsible, at least in part, to elevated high blood pressure. The decreased ability of the neonatal LV to cope with sustained exposure to increased afterload is a concern, but data are limited. Leipälä et al. (36) demonstrated higher LV stroke volume immediately after birth but altered capacity to increase further LVO during the first postnatal week compared to healthier AGA. High blood pressure and, more recently, increased carotid-media thickening are demonstrable risk factors for adverse cardiovascular outcomes in adult life (52). These findings, along with arterial wall remodeling, raised SVR and higher blood pressure support the Barker hypothesis of *in utero* programming of chronic diseases (53). The long term ramifications of altered BP profiles in childhood and beyond requires prospective evaluation.

During fetal life the RV output bypasses the lungs through the DA into the descending aorta (54). In the setting of FGR, abnormalities of cardiac shape and poor RV contractility have been noted (55). After birth, echocardiography studies during the postnatal transition showed higher RV vs. LV mass index (56) and higher RVO vs. LVO (57), which underline the dominant role of RV in the transition. In a prospective observational study of healthy term infants, Jain et al. (15) demonstrated a delayed increase in RV, but not LV, performance (FAC-3C and FAC-4C) from 5 h age to 35 h age. This observation of differential response to changes in loading conditions may reflective intrinsic RV potential or paradoxical intolerance of the RV to the increase in SVR while the PDA remains open. Regardless, these findings suggest an intrinsic vulnerability of the RV during the transitional period which may be exaggerated in the setting of acute pulmonary hypertension (58) or hypoxic ischemic encephalopathy (59). Of importance, the newly born SGA infant may need a longer time to adapt to extrauterine environment and may have heightened RV vulnerability which justifies future studies with long term follow up to characterize child and adulthood repercussions. The transitional changes in RV systolic performance are consistent with observational data from Sehgal et al. (41) in a preterm cohort of SGA evaluated 5 h after birth. The higher rate of abnormal TAPSE vs. RV FAC threshold requires additional consideration. As heart dimensions are likely to be smaller in IUGR infants, these data suggest the need to index TAPSE or develop unique normative datasets for this population. Due to advances in neonatal echocardiography, the clinical relevance of the impaired RV systolic performance in neonatal disease is becoming more apparent. Human studies of asphyxiated term newborns demonstrated that RV dysfunction was an independent predictor of death or severity of brain injury (59). The authors demonstrated an important relationship of impaired RV performance to abnormal cerebral hemodynamics, higher SNAPPE-II scores, and greater encephalopathy. In addition, survivors were more likely to have abnormal neurodevelopmental outcome at 2 years (60). Therefore, it is plausible that early recognition of the RV impaired function in early stage of the transitional period may play an important role in timely management of these vulnerable population and a modifiable outcome.

Strengths of our study is that study subjects were included in fetal life, they had a close prenatal care and monitoring until birth and followed up during the immediate transition to extrauterine life up to 48 h age when the major changes in cardiopulmonary physiology occur. Second, all variables were recorded prospectively in a cohort of infants, detected to be SGA before 32 weeks of gestation, born at term and matched according to mode of delivery with healthy term infants. Furthermore, primary, and secondary outcomes were evaluated based on comprehensive TnECHO, which is an essential tool to facilitate diagnostic precision and to provide physiologically appropriate treatment choices (61). There are, however, some important limitations. *First*, spectral Doppler is operator dependent (62, 63). However, spectral Doppler analysis is known to be reliable in infants compared to other invasive methods in measuring the cardiac output (64). This work provide hypothesis generating data, with a small cohort and with no subsequent follow up of hemodynamic variables. *Second*, the relationship to adverse neonatal health outcomes and whether the changes noted are permanent remains unknown. Third, advanced imaging technique i.e., Tissue Doppler (would have provide much needed information on diastolic disfunction) and Speckle tracking echocardiography (an angle independent technique) or a combination of high frame color Doppler data with speckle tracking analysis blood speckle imaging (BSI) were not used in this study.

## Conclusions

Characterization of the cardiovascular adaptative changes during transitional period is important among SGA infants for appraising disease susceptibility. Our study showed that SGA infants had higher PVR, lower RV performance and impaired LV diastolic function. The clinical relevance and impact of these changes to hemodynamic disease states during the postnatal transition requires prospective investigation.

## Data Availability

The raw data supporting the conclusions of this article will be made available by the authors, without undue reservation.
