# Supplementary material for: Comparative evaluation of echocardiography indices during the transition to extrauterine life between small and appropriate for gestational age infants
Source: Front Pediatr. 2023 Jan 16;10:1045242. doi: 10.3389/fped.2022.1045242 (PMC9884809; doi:10.3389/fped.2022.1045242)
Supplement: Supplementary file 1 [file Table1.docx]

**Table 1**. List of echocardiography indices used in this study

| **Echocardiography-derived indices** | |
| --- | --- |
| Right ventricular function | Systolic function  Tricuspid annular plane systolic excursion (TAPSE)  Fractional area change (FAC) 3- chamber apical view  FAC= (RVEDA-RVESA)/ RVEDA X 100  Diastolic function  Tricuspid valve inflow (E, A, E/A) |
| Left ventricle function | Systolic function  Ejection fraction (LVEF) - Simpson biplane method  Fractional shortening (LVSF)  LVSF (%) = [(LVEDD-LVESD)/LVEDD] X 100%  Diastolic function  Isovolumic relaxation time (IVRT)  Mitral valve inflow (E, A, E/A) |
| Pulmonary blood flow | RVO (ml/kg/min) = [VTI (cm) x π x (D/2)^2^ x HR] indexed to weight |
| Systemic blood flow | LVO (ml/kg/min) = [VTI (cm) x π x (D/2)^2^ x HR] indexed to weight |
| Pulmonary vascular resistance | Right ventricular ejection time (RVET) and Pulmonary artery acceleration time (PAAT) were measured from the pulse-wave Doppler tracing of the main pulmonary artery obtained from the parasternal long-axis view of the right ventricular outflow tract with a 2-mm wide Doppler gate placed in the middle of the main pulmonary artery at the level of the pulmonary valve.  PVRi=RVET/PAAT |
| Systemic vascular resistance | Left ventricle ejection time (LVET)  Aortic acceleration time (AoAT)  SVRi=LVET/AoAT |
| LV/RV interaction | Interventricular septal motion in systole |
| Shunts | Patent foramen ovale (PFO)  Patent ductus arteriosus (PDA) |
| Celiac/Superior mesenteric artery | Peak systolic (PS) velocity (point), Peak end-diastolic (ED) velocity(point), and Mean Velocity (trace) |
| Middle cerebral artery | Peak systolic (PS) velocity (point), Peak end-diastolic (ED) velocity(point), and Mean Velocity (trace) |

**Table 2**. Effect of group and time on systemic blood flow velocities

|  | **SGA Group**  **n=18** | | **AGA Group**  **n=18** | | **P value (Time)*** | **P value (Group)^#^** |
| --- | --- | --- | --- | --- | --- | --- |
|  | *Echo 1* | *Echo 2* | *Echo 1* | *Echo 2* |  |  |
| CA PS, cm/s | 56.1 (20.9) | 63.3 (23.1) | 67.1 (20.7) | 70.3 (18.7) | 0.17 | 0.13 |
| CA ED, cm/s | 16.5 (7.3) | 18.1 (9.8) | 20.4 (9.2) | 20.6 (8.3) | 0.62 | 0.18 |
| CA Mean, cm/s | 17.3 (16.1) | 18.4 (16.2) | 26.9 (16.5) | 27.1 (16.5) | 0.76 | 0.77 |
| CA RI | 0.6 (0.1) | 0.6 (0.2) | 0.6 (0.1) | 0.7 (0.1) | 0.39 | 0.18 |
| CA PI | 1.1 (1.1) | 1.1 (0.9) | 1.3 (0.5) | 1.4 (0.5) | 0.64 | 0.45 |
| SMA PS, cm/s | 40.6 (32.1) | 50.3 (39.4) | 51.2 (40.8) | 65.3 (46.7) | 0.07 | 0.28 |
| SMA ED, cm/s | 8.7 (8.5) | 12.2 (12.1) | 12.1 (10.7) | 12.4 (10.2) | 0.22 | 0.57 |
| SMA Mean, cm/s | 8.7 (11.1) | 11.1 (15.3) | 17.4 (21.6) | 18.1 (19.3) | 0.52 | 0.14 |
| SMA RI | 0.5 (0.3) | 0.5 (0.3) | 0.5 (0.3) | 0.5 (0.3) | 0.49 | 0.86 |
| SMA PI | 1.1 (1.4) | 1.1 (1.2) | 1.1 (0.9) | 1.4 (1.1) | 0.62 | 0.61 |
| MCA PS, cm/s | 29.2 (10.7) | 37.6 (14.6) | 39.4 (11.5) | 42.4 (13.9) | 0.06 | 0.01 |
| MCA ED, cm/s | 8.3 (5.1) | 11.1 (5.3) | 11.6 (5.1) | 11.9 (4.9) | 0.19 | 0.11 |
| MCA Mean, cm/s | 16.6 (7.5) | 20.3 (8.9) | 21.3 (6.6) | 21.1 (6.5) | 0.24 | 0.17 |
| MCA RI | 0.6 (0.2) | 0.6 (0.1) | 0.7 (0.1) | 0.7 (0.1) | 0.89 | 0.62 |
| MCA PI | 1.1 (0.8) | 1.1 (0.7) | 1.3 (0.2) | 1.4 (0.4) | 0.91 | 0.07 |

CA= celiac artery; SMA= superior mesenteric artery; MCA=middle cerebral artery; PI=pulsatility index; RI= resistivity index; ED= end diastolic; PS= peak systolic. p values are the results of general linear model repeated measures. NS= p>0.05. Values are presented as mean and standard deviation.

*P value (time) represents the significance of changing values over the two different time points

# P value (group) represents the significance of the difference between the two groups
